# Exploring the levels of persistent organic pollutants in umbilical cord blood and their connection to gestational age and birth weights in Şanlıurfa, Turkey

**DOI:** 10.1186/s12884-024-06677-8

**Published:** 2024-07-25

**Authors:** Sıddika Songül Yalçin, Bülent Güneş, Kalender Arikan, Orhan Balçik, Özcan Kara, Suzan Yalçin

**Affiliations:** 1 Department of Pediatrics, Faculty of Medicine, Hacettepe University, Ankara, Turkey; 2 Child Health and Disease Service, Şanlıurfa Training and Research Hospital, Şanlıurfa, Turkey; 3 Department of Biology Education, Faculty of Education, Hacettepe University, Ankara, Turkey; 4 Pesticide Research and Reference Laboratory, Hacettepe University, Ankara, Turkey; 5 Gynecology and Obstetrics Clinic, Private Şan Med Hospital, Şanlıurfa, Turkey; 6 Department of Food Hygiene and Technology, Faculty of Veterinary Medicine, Selcuk University, Konya, Turkey

**Keywords:** Polychlorinated biphenyls, Organochlorine pesticides, Umbilical cord blood, Appropriate for gestational age

## Abstract

**Background:**

Controversy surrounds the impact of persistent organic pollutants (POPs) on fetal development. This study aimed to investigate levels of polychlorinated biphenyls (PCBs) and organochlorine pesticides (OCPs) in umbilical cord blood from Şanlıurfa mothers in Turkey, exploring associations with gestational age and birth weight.

**Methods:**

Participants included voluntary mothers pregnant with a single fetus, providing details on maternal factors. Cord blood samples were collected immediately after delivery. Samples were extracted with a modified QuEChERS method, and OCPs (17 pesticides) and PCBs (11 congeners) compound levels were analyzed with a gas chromatograph/mass spectrometry. Detection frequencies and levels of POPs by single pollutant type and pollutant groups were calculated and compared according to gestational duration and birth weight. We used partial least squares discriminant analysis to identify the key chemicals and distinguish their respective statuses.

**Results:**

Among 120 infants, 35 were preterm but appropriate for gestational age, 35 were term but small for gestational age (SGA), and 50 were term and appropriate for gestational age (AGA). Beta HCH, Oxy-Chlordan, and PCB 28, were not detected in cord blood samples. Half of the samples contained at least 4 types of OCPs, with a median OCP level of 38.44 ng/g. Among the DDT, 2,4’-DDE was found at the highest concentration in cord plasma samples. The PCB congeners with a frequency exceeding 50% were ranked in the following order: 151, 149, 138, 146. The median level of ∑PCBs was 5.93 ng/g. Male infants born at term with SGA status exhibited lower levels of ∑DDTs, ∑OCPs compared to male infants born preterm or at term with AGA status. Di-ortho-substituted PCBs and hexachlorinated PCBs were higher in male infants born at term with SGA status than male infants born preterm with AGA status.

**Conclusion:**

Overall, exposure to DDT and PCBs demonstrates varying effects depending on gestational duration and birth weight, with exposure levels also differing by gender. This underscores the necessity for studies across diverse populations that investigate the combined effects of multiple pollutant exposures on gestational age, birth weight, and gender simultaneously.

**Supplementary Information:**

The online version contains supplementary material available at 10.1186/s12884-024-06677-8.

## Introduction

Persistent organic pollutants (POPs) represent a class of chemicals characterized by their propensity for biological accumulation, persistence in the environment, and harmful effects on living organisms. Notable examples of POPs include polychlorinated biphenyls (PCBs) and organochlorine pesticides (OCPs). OCPs were widely used as synthetic insecticides from the 1940s to the 1960s, while PCBs found extensive application in dielectric fluids and transformers. Recognizing the detrimental impact of these substances on both the environment and human health, a global initiative took shape, leading to the establishment of the Stockholm Convention in 2001. This international treaty aimed to mitigate environmental levels of POPs and restrict their emissions. Despite these efforts, the banned pollutants persist in nature, underscoring the challenges associated with their elimination [1–5]. PCBs, owing to their lipophilicity and prolonged half-lives, exhibit a remarkable ability to bioaccumulate in adipose tissue. The concentration of PCBs in umbilical cord plasma serves as a reliable indicator of prenatal exposure to these pollutants. Furthermore, these compounds can traverse the placental barrier, transferring to the fetus, and are subsequently excreted into maternal milk, resulting in potential exposure for infants. The continued presence of these banned substances emphasizes the importance of ongoing monitoring, research, and regulatory measures to address and mitigate the enduring environmental and health impacts associated with POPs.

The developing embryo and fetus are particularly vulnerable to external factors during pregnancy, and birth weight stands out as a crucial indicator of fetal growth [6–14]. Various factors, including genetics, maternal nutrition, placental circulation, and exposure to pollutants, can significantly influence this key metric [15, 16]. The impact of some POPs on fetal development is a subject of ongoing debate and research. Existing epidemiological studies have predominantly focused on exploring associations between single pollutant exposures and birth weight or other fetal growth measures, including birth length, head circumference, and the likelihood of being small for gestational age. Fetuses exposed to high levels of OCPs face increased risks of various health complications, including low birth weight, congenital malformations, infections, stillbirths, and the potential for long-term disease risk through developmental programming [15, 17]. However, 4,4′-DDE has been linked to increased infant growth [18], introducing a level of complexity and controversy to the understanding of the effects of specific POPs, On the other hand, certain PCBs exhibit minimal or no associations with birth weight [15], adding further nuance to the intricate relationship between pollutants and fetal development. PCBs have been specifically associated with reduced fetal growth parameters, such as abdominal circumference or femur length [19]. The results are inconsistent across various studies, with some reporting no significant associations [20, 21]. This inconsistency highlights the complexity of understanding the nuanced relationship between specific pollutants and their impact on fetal development, emphasizing the need for continued research and a comprehensive approach to assessing the potential risks associated with these environmental contaminants.

Recognizing the limitations of investigating associations with single pollutant exposures, there is a growing understanding that real-life situations involve complex and simultaneous exposure to numerous pollutants throughout an individual's lifetime [22]. This recognition has prompted a shift in research focus towards better assessing the risks associated with mixtures of pollutants and understanding the cumulative effects of multiple exposures and stressors [22]. Recent biomonitoring studies have responded to this need by examining the associations of multiple exposures with fetal growth [23, 24]. This more comprehensive approach allows researchers to explore the intricate interactions and potential synergistic or antagonistic effects that may arise from exposure to a mixture of pollutants. By moving beyond the study of individual pollutants and considering the broader context of multiple exposures, these biomonitoring studies contribute to a more holistic understanding of the environmental factors influencing fetal growth. This shift in research paradigm aligns with the increasing awareness of the complexity of environmental exposures and their potential health impacts, emphasizing the importance of adopting a more integrated and nuanced approach to assess the risks associated with the multitude of pollutants that individuals may encounter over their lifetimes.

In Turkey, there is a scarcity of studies in breast milk, blood sample of newborn examining the presence of POPs [25–30]. Existing research in blood samples of newborns has predominantly concentrated on pollutant concentrations and types [25]. Recognizing the controversial nature of the effects of POPs on fetal development and their potential impact on gestational development in newborns [11–15, 31], the primary objective of this study is to investigate the levels of PCBs and OCPs in umbilical cord blood and potential associations between gestational age and birth weight. By focusing on these specific outcomes, the research aims to shed light on the potential impact of these pollutants on the duration of pregnancy and the weight of newborns at birth.

If the study reveals any significant relationship between concentrations of certain pollutants and gestational age or birth weight, it would open up the possibility of implementing preventive measures for pregnant women. This may involve targeted efforts to minimize exposure pathways for pregnant women, ultimately contributing to improved maternal and fetal well-being. The findings from this study have the potential to inform public health policies and guidelines, offering concrete steps to mitigate the impact of POPs on gestational outcomes in the Turkish population.

## Methods

### Sampling areas, program, and preparation

Şanlıurfa, with approximately 2.17 million inhabitants, stands as one of Turkey's most densely populated cities. According to data from the Turkish Statistical Institute, the total fertility rate in Turkey was 1.62 children in 2022, while Şanlıurfa exhibited the highest rate at 3.59 children [32]. This demographic insight highlights the city's distinctiveness in terms of population dynamics and childbirth.

The study protocol received approval from the Harran University Clinical Research Ethics Committee (HRÜ/23.08.05). The study encompassed pregnant women admitted to the Obstetrics Center of Şan Hospital for delivery in 2017 as a part of ‘Urfa Child Cohort Survey’ [33]. Prior to participation, pregnant women were provided with detailed information about the study, and their voluntary involvement was secured through written informed consent. Exclusion criteria encompassed pregnancies involving twins or triplets.

Upon hospital admission, all participating mothers were requested to complete a study form, providing information such as maternal age, height, pre-pregnancy weight, gestational weight gain, and sociodemographic details. Umbilical cord blood samples were promptly collected in EDTA-containing tubes immediately after delivery. These samples were obtained from the segment of the umbilical cord adjacent to the placenta, post-clamping and cutting.

Prematurity, defined as gestational age less than 37 weeks, served as a criterion for categorizing newborns in this study. Utilizing the growth curve database, infants were further classified into distinct groups: small for gestational age (SGA), denoting birth weight below the tenth percentile; and appropriate for gestational age (AGA), indicating birth weight falling between the tenth and ninetieth percentiles for both genders [34].

Prepregnancy body mass index (BMI) was calculated. To examine maternal weight gain during pregnancy, a standardized classification was employed, considering low, appropriate, and high categories based on prepregnancy weight. This classification system, outlined in accordance with established standards, enhances the precision of assessing gestational weight gain and its potential implications [35].

### Sampling and instrumental analysis

For the determination of PCB congeners and OCPs, plasma derived from cord blood centrifugation was used. After centrifugation, the samples were stored at -20 °C until further analysis. The collected plasma volume was approximately 1.5 ml.

The suite of OCPs scrutinized included the α-, β-, γ-, and δ-isomers of hexachlorocyclohexane (HCH), dichlorodiphenyltrichloroethane (DDT) and its derivatives (2,4′-DDE, 2,4′-DDD, 2,4′-DDT, 4,4′-DDE, 4,4′-DDD, and 4,4′-DDT), hexachlorobenzene (HCB), chlordanes (CHLs) encompassing chlorothalonil, oxychlordane, trans-chlordane, and cis-chlordane, pesticides derived from hexachlorocyclopentadienes (HCCPDs) (α- and β-endosulfan; endosulfan-sulfate; and cis- and trans-isomers of heptachlor, aldrin, endrin, dieldrin), and dicofols (dicofol and 2,4′-dicofol). The targeted PCB congeners (IUPAC numbers) were 28, 52, 95, 99, 101, 105, 110, 118, 138, 146, 149, 151, 153, 156, 170, 177, 180, 183, and 187. Triphenyl phosphate served as the internal standard for calibration purposes, and all standards were procured from Dr. Ehrenstorfer’s Laboratories in Augsburg, Germany, at a concentration of 10 ng/μL in iso-octane.

### Cord plasma extraction and clean-up

For the extraction procedure of umbilical plasma, a modified QuEChERS (Quick, Easy, Cheap, Effective, Rugged, and Safe) method, adapted from previous studies, was employed [36, 37]. In a 15 mL centrifuge tube, 2.0 mL of acetonitrile (1.0% Acetic Acide) containing triphenyl phosphate as an internal standard (IS) was added to 1 mL of plasma sample. Subsequently, 400 mg MgSO_4_ anhydrous and 100 mg NaCl (Q-sep QuEChERS Extraction Salt Packets from Restek – U.S) was introduced into the 15 mL centrifuge tube, followed by a 5-min shaking period using a shaker. The resulting mixture was then centrifuged at 3500 rpm for 5 min. After centrifugation, all of the supernatant was transferred to a 2.5 mL microcentrifuge tube containing 150 mg MgSO_4_, 50 mg PSA, and 50 mg C18-EC (Q-sep QuEChERS dSPE from Restek – U.S). These tubes underwent a 1-min shaking step using a shaker, followed by centrifugation at 3500 rpm for 5 min. For further processing, 1.0 mL of the extract was withdrawn from the tubes and added to a 2.0 mL autosampler vial after filtration with a 0.20 μm pore size sterile filter (Minisart NML, CA).

### Gas chromatograph/mass spectrometry (GC/MS) conditions

Chromatographic analysis was conducted using a Shimadzu QP2010Ultra GC–MS (Shimadzu, Japan), equipped with a capillary column measuring 30 m in length, 0.25 mm in inner diameter, and coated with a 0.25 μm thick film (Rxi-5Sil MS, Restek Corporation, USA). The injection was performed in splitless mode an injector temperature set at 270°C. Helium was used as the carrier gas at a flow rate of 1.48 mL/min. Oven temperature was programmed as follows: initially held at 120 °C hold for 2 min, then ramped up to 180°C at the rate of 15°C/min, and finally increased by 6°C/min to reach 300°C (maintained for 4 min.). Electron impact ionization was employed as the ion source, with the ion source temperature set to 230°C and the transfer line temperature maintained at 270°C. A selected ion monitoring (SIM) program was developed for the detection of selected ions corresponding to PCBs and OCPs (Supplementary Table).

### Quality assurance

Validation of the method was performed with respect to limit of detection (LOD), limit of quantification (LOQ), precision, recovery, linearity, and measurement uncertinity (Supplementary Table). Calibration in spiked blood samples for the quantification of PCB and OCP solutions in iso-octanes were established with two eight-point concentration of 0.1, 0.5, 1.0, 2.0, 5.0, 10.0, 50.0 and 100.0 ng/g. Linearity (r^2^ ≥ 0.99) was consistently observed across the tested ranges. Intra- and inter-day precisions were evaluated by carrying out the five injections of PCBs and OCPs a concentration of 10 ng/g in blood. Recovery study was carried out at three different concentration levels such as 1.0, 5.0 and 10.0 ng/g in blood. Mean value of key performance parameters, including r^2^ (0.99 ± 0.0004), percent recovery (100.10 ± 3.16%), LOD (0.19 ± 0.06 ng/g), LOQ (0.58 ± 0.19 ng/g), and measurement uncertinity (0.067 ± 0.23 ng/g) were determined through linear regression of the multi-level calibration (Supplementary Table). All analyses were meticulously carried out at the Hacettepe University Pesticide Research and References Laboratory, ensuring high standards of precision and reliability in the results. The reporting of POPs in cord blood plasma was based on wet weight, denoted in nanograms per gram (ng/g).

Notably, beyond the analysis of individual PCBs (11 congeners) and OCPs (17 pesticides), we extended our investigation to encompass aggregated measures. These included the sum of concentrations for HCH isomers (∑HCH), DDT isomers/metabolites (ΣDDT), total chlorinated hydrocarbons (∑CHLs), total hexachlorocyclopentadienes (∑HCCPDs), overall organochlorine pesticides (∑OCPs), and the sum of polychlorinated biphenyls (∑PCB).

We categorized the PCB congeners based on previously published classification groupings from studies by Wolff et al. [38], McFarland and Clarke [39], Cooke et al. [40], and Kannan and Petrick [41], as well as a category labeled 'Combined' [42]. Additionally, we considered the degree of ortho-substitution and total chlorination degree. We measured concentrations of three monoortho-substituted PCBs (congeners 28, 105, 118), nine di-ortho-substituted PCBs (congeners 52, 99, 101, 110, 138, 146, 153, 170, 180), and six tri-ortho-substituted PCBs (congeners 95, 149, 151, 177, 183, 187). Studied PCBs were pentachlorinated (95, 99, 101, 105, 110, 118), hexachlorinated (138, 146, 149, 151, 153), and heptachlorinated (170, 177, 180, 183, 187) [43]. This comprehensive approach allowed for a thorough assessment of contaminant levels in the umbilical cord blood samples.

### Statistical analysis

Our study used IBM-SPSS Statistics version 26.0 (SPSS Inc., Chicago, IL, USA) for both database management and statistical analysis. The concentrations of DDT and its metabolites, HCH isomers, all other OCPs, and PCBs in umbilical cord blood samples were characterized using several key parameters. These parameters include the LOD measured in ng/g, distribution of frequencies, percentage of values above the LOD, as well as values at the 10th, 25th, 50th, 75th, and 90th percentiles.

OCPs and PCB congeners having detection rates between 10 and 90% of samples were analysed and compared. For variables detected at 90% or higher, in cases under the detection threshold (DT), a square root of 2 level of the limit of detection (LOD) value was assigned, and average/median values were calculated. Categorical variable relationships were assessed using the chi-square test, while the Kolmogorov–Smirnov test was employed to evaluate normality for continuous variables. Skewed data were subjected to Kruskal–Wallis tests, and normally distributed variables underwent One-way ANOVA.

Generalized Linear Models were used to investigate differences in pollutant levels (OCPs, DDTs, and PCBs) relative to gestational duration and birth weight categories (preterm + AGA, term + SGA, term + AGA), and confounding factors such as mother’s age (years), mother’s education (< 12 years, ≥ 12 years), mother’s occupation (housewife, with job), BMI before pregnancy (kg/m^2^), weight gain during pregnancy (low, appropriate, high), smoke exposure during gestational period (absence, presence), type of birth (vaginal delivery, cesarean delivery), parity (first child, ≥ 2nd children), gender (male, female) with an identity link and pairwise comparisons with LSD. Mean values with 95% Wald Confidence Intervals were reported. Statistical significance was defined as a two-tailed probability level of < 0.05.

The studied chemicals were normalized with autoscaling (mean-centered and divided by the standard deviation of each variable) in MetaboAnalyst5.0 software. Partial least squares discriminant analysis (PLS-DA) was applied in the encounter of many possible correlated predictor chemicals (X) in a matrix of responses [y: preterm + AGA, term + SGA, term + AGA] to understand which chemicals carry the class separating information and Variable importance in projection (VIP) scores of the chemicals were measured.

## Results

### General characteristics

Out of 145 births, three were multiple pregnancies, and 120 of them consented to participate in the study. Table 1 provides the characteristics of both mothers and newborns. The average age of pregnant women was 26.4 ± 4.8 years (range: 16–39), with 15% of them employed. The mean pre-pregnancy BMI was 25.6 kg/m^2^, and 19.2% were classified as obese. The average weight gain during pregnancy was 12.2 kg. One-fourth of the participants achieved normal weight gain during pregnancy. Some mothers experienced threats of miscarriage (*n* = 7), anemia (*n* = 3), gestational diabetes (*n* = 4), and hypertension (*n* = 3). Nearly half of them had exposure to tobacco smoke. A majority of the mothers underwent cesarean delivery (73.3%), and 65.0% were multiparous.

**Table 1:** General characteristics of mother-infant pairs, *n* = 120

	% or Mean ± SD (min–max)
Mother’s age, yrs	26.4 ± 4.8 (16–39)
Mother’s age < 25 yrs	35.0
Mother’s education ≥ 12yrs	35.0
Mothers with job	15.0
Having smoke exposure or smoking	50.8
Prepregnancy BMI, kg/m^2^	25.6 ± 4.9 (12.9–40.7)
≥ 30 kg/m^2^ (obese)	19.2
Weight gain during pregnancy	12.2 ± 6.6 (2–25)
Low	33.3
Normal	26.7
High	40.0
Parity, first child	35.0
Sex, male	53.3
Birth type, ceserean delivery	73.3
Birth weight, kg	2.91 ± 0.57 (1.4–4.2)
Gestational duration, weeks	37.8 ± 2.1 (30–41)

The newborns had an average weight of 2.91 kg, with a gestational duration of 37.8 weeks, and half of them were male. Among the infants, 35 were preterm but AGA, 35 were term but SGA, and 50 were term and AGA. Notably, one infant had cryptorchidism, and another had a pilonidal sinus.

### Cord plasma OCPs and PCBs levels

Table 2 presents the concentrations of OCPs and PCBs assessed in umbilical cord plasma, providing a summary based on medians, maximum values, and percentiles including 10th, 25th, 75th, and 90th. Notably, certain contaminants, including beta HCH, oxy-Chlordan, and PCB 28, were not detected in cord blood samples.

**Table 2:** The percentage (%) of cases with cord blood levels of persistent organic pollutants above the limit values, as well as the levels (ng/g) detected at the 10th, 25th, 50th, 75th, and 90th percentiles, *n* = 120

	samples ≥ LOD	Levels at percentiles, ng/g					Max
	%	10th	25th	50th	75th	90th	
alpha-HCH	28.3	-	-	-	3.34	3.99	6.98
beta-HCH	0.0	-	-	-	-	-	-
delta-HCH	5.8	-	-	-	-	-	6.94
gamma-HCH	37.5	-	-	-	3.68	4.65	6.68
∑**HCH**	58.3	-	-	3.42	4.60	7.04	13.94
2,4’-DDD	17.5	-	-	-	-	4.54	5.93
4,4’-DDD	14.2	-	-	-	-	3.90	7.64
2,4’-DDE	79.2	-	5.19	23.61	29.64	34.04	46.13
4,4’-DDE	9.2	-	-	-	-	-	5.49
2,4’-DDT	11.7	-	-	-	-	4.58	7.53
4,4’-DDT	40.8	-	-	-	5.13	5.48	35.65
∑**DDTs**	96.7	3.92	13.26	27.25	33.64	39.47	46.85
oxy-Chlordan	0.0						
cis-Chlordan	23.3	-	-	-	-	7.49	7.71
trans-Chlordan	23.3	-	-	-	-	9.53	9.89
chlorothalonil	19.2	-	-	-	-	7.90	7.94
∑**CHLs**	56.7	-	-	6.50	9.27	9.87	17.16
Aldrin	8.3	-	-	-	-	-	19.42
Endrin	6.7	-	-	-	-	-	28.36
Heptachlor	6.7	-	-	-	-	-	50.51
Endosulfan-sulfate	5.8	-	-	-	-	-	12.64
∑**HCCPDs**	25.8	-	-	-	2.7	12.5	50.51
Total Dicofol (2,4’)	8.3	-	-	-	-	-	8.75
HCB	19.2	-	-	-	-	5.87	34.56
∑**OCPs**	99.2	10.37	21.65	38.44	50.56	63.10	104.53
PCB 28	0.0						
PCB 52	5.0	-	-	-	-	-	0.99
PCB 95	6.7	-	-	-	-	-	1.30
PCB 99	11.7	-	-	-	-	0.65	2.42
PCB 101	10.0	-	-	-	-	0.20	2.36
PCB 105	15.0	-	-	-	-	0.63	1.23
PCB 110	9.2	-	-	-	-	-	1.54
PCB 118	25.0	-	-	-	0.51	1.20	1.53
PCB 138	59.2	-	-	1.50	1.84	2.14	3.22
PCB 146	53.3	-	-	1.00	1.62	1.66	2.17
PCB 149	60.0	-	-	1.30	1.74	1.82	2.87
PCB 151	66.7	-	-	2.01	2.25	2.47	2.82
PCB 153	30.0	-	-	-	1.25	1.62	2.57
PCB 170	20.0	-	-	-	-	1.38	1.68
PCB 177	20.8	-	-	-	-	1.09	1.42
PCB 180	6.7	-	-	-	-	-	1.00
PCB 183	5.8	-	-	-	-	-	0.68
PCB 187	7.5	-	-	-	-	-	1.13
∑**PCBs**	98.3	3.44	4.56	5.93	7.78	9.00	11.40

*CHLs* chlordanes, *DDD* dichlorodiphenyldichloroethane, *DDE* dichlorodiphenyldichloroethylene, *DDT* dichlorodiphenyltrichloroethane, *HCB* hexachlorobenzene, *HCCPDs* hexachlorocyclopentadienes, *HCH* hexachlorocyclohexane, *OCPs* organochlorine pesticides, *PCBs* polychlorinated biphenyls

Total HCH was detected in 58.3% of the samples, primarily contributed by gamma-HCH and alpha-HCH. Among the DDT, 2,4’-DDE was found at the highest concentration in cord plasma samples. Either 4,4’-DDT or 4,4’-DDE was detected in the blood samples of 57 cords. Both 4,4’-DDT and 4,4’-DDE were found in four samples, while only 4,4’-DDE was found in seven samples, and only 4,4’-DDT was found in 46 samples. The 4,4’-DDT/4,4’-DDE ratio was above 1. Total CHLs were present in 56.7% of the samples, predominantly consisting of cis-Chlordan and trans-Chlordan. In 99.2% of the samples, at least one OCP was identified. In one case, 9 OCPs were detected, 8 in another, 7 in four samples, and 6 OCPs in 11 samples. Approximately half of the samples contained at least 4 OCPs, with a median ∑OCP level of 38.44 ng/g.

In umbilical cord samples, at least one PCB congener was detected in 98.3% of cases, with up to 8 congeners identified simultaneously among the 18 studied. Specifically, two samples exhibited 8 PCB congeners, 4 samples had 7 congeners, and 6 samples contained 19 congeners. The predominant composition across samples featured two congeners in 29.1% of cases and three congeners in 25.7%. PCB 151 emerged as the most prevalent congener, found in 66.7% of samples, with a median value of 2.25 ng/g. Notably, PCB 28 was not detected. The PCB congeners with a frequency exceeding 50% were ranked in the following order: 151, 149, 138, 146. The median level of ∑PCBs was 5.93 ng/g.

### Differences in OCPs and PCBs according to gestational age and birth weight

**Fig. 1:**
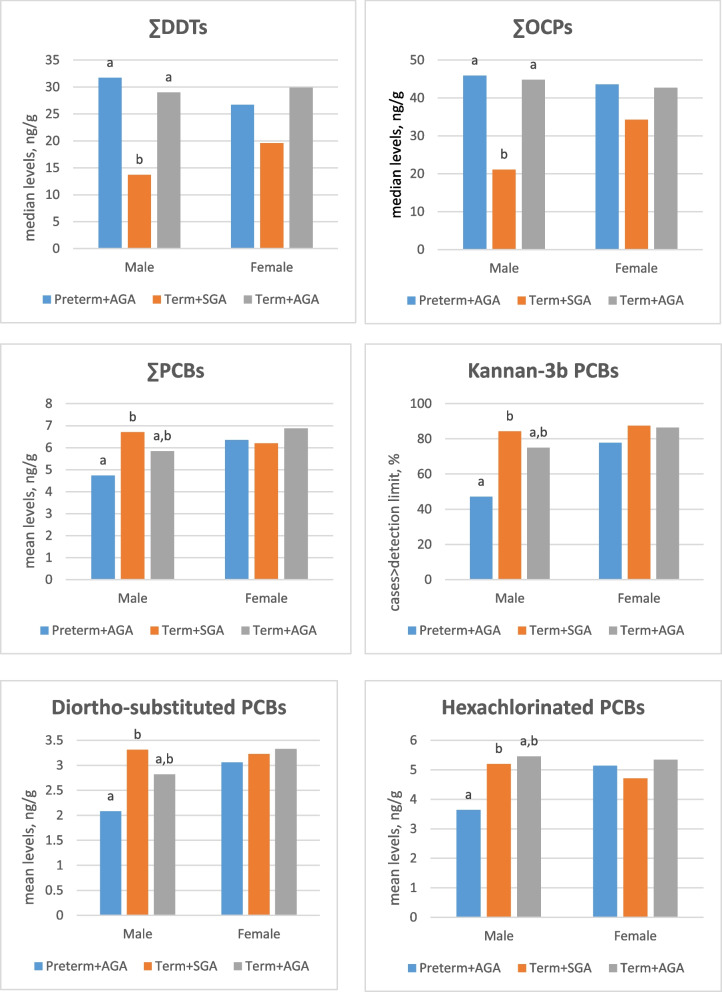
Levels of certain persistent organic pollutants in cord blood according to gender [AGA: appropriate for gestational age; DDT: dichlorodiphenyltrichloroethane; OCPs: organochlorine pesticides; PCBs: polychlorinated biphenyls; SGA: small for gestational age]

Differences in detection frequencies were observed for gamma-HCH among the groups (*p* = 0.011), with the lowest frequencies found in the term + SGA group (Table 3). The detection frequency of ∑HCH was the highest in term + AGA cases (*p* = 0.033). The detection frequency of 4,4’-DDT was also lowest in the preterm + AGA group (*p* = 0.032). Significant variations in the median levels of total DDTs and total OCPs were noted (*p* = 0.001 for each), with the lowest levels observed in the term + SGA group (*p* < 0.05). The median levels of ∑DDTs and ∑OCPs were lowest in term + SGA group. There is a difference according to gender, and this relationship was observed only in the cord blood of male infants (Fig. 1).

**Table 3:** The percentages of cases above the detection limits or median values of OCPs and PCBs in cord blood according to gestational duration and birth weight

	Preterm + AGA (*n* = 35)	Term + SGA (*n* = 35)	Term + AGA (*n* = 50)	p
Alpha-HCH*	17.1	25.7	38.0	0.101
Gamma-HCH*	42.9^a^	17.1^b^	48.0^a^	0.011
∑HCH*	51.4^ab^	45.7^b^	72.0^a^	0.033
2,4’-DDD*	11.4	22.9	18.0	0.450
4,4’-DDD*	5.7	14.3	20.0	0.178
2,4’-DDE*	80.0^ab^	65.7^b^	88.0^a^	0.045
2,4’-DDT*	11.4	8.6	14.0	0.744
4,4’-DDT*	22.9^a^	51.4^b^	46.0^b^	0.032
∑DDTs**	30.3 (7.6–36.6)^a^	15.9 (5.7–27.2)^b^	29.6 (22.8–35.2)^a^	0.001
**Male**	31.7 (14.8–38.3)^a^	13.7(4.3–24.5)^b^	29.0 (22.0–36.2)^a^	0.004
**Female**	26.7 (7.6–35.7)	19.6 (10.1–29.2)	29.9 (23.4–34.1)	0.143
cis-Chlordan*	22.9	22.9	24.0	0.989
trans-Chlordan*	28.6^a^	8.6^b^	30.0^a^	0.049
chlorothalanil*	25.7	20.0	14.0	0.397
∑CHls*	68.6	45.7	56.0	0.154
∑HCCPDs*	28.6	20.0	28.0	0.644
HCB*	20.0	11.4	24.0	0.346
∑OCPs**	44.7 (15.8–55.4)^a^	27.8 (16.3–37.8)^b^	44.0 (33.4–54.2)^a^	0.001
**Male**	45.9 (24.9–58.4)^a^	21.1 (13.1–30.2)^b^	44.8 (34.8–61.5)^a^	< 0.001
**Female**	43.6 (14.7–54.5)	34.3 (25.9–43.4)	42.7 (30.7–50.2)	0.524
PCB 99*	8.6	17.1	10.0	0.477
PCB 101*	11.4	14.3	6.0	0.431
PCB 105*	8.6	17.1	18.0	0.446
PCB 118*	31.4	20.0	24.0	0.531
PCB 138*	45.7	65.7	64.0	0.155
PCB 146*	48.6	60.0	52.0	0.613
PCB 149*	57.1	62.9	60.0	0.888
PCB 151*	57.1	77.1	66.0	0.205
PCB 153*	28.6	31.4	30.0	0.967
PCB 170*	14.3	22.9	22.0	0.601
PCB 177*	17.1	22.9	22.0	0.812
∑PCBs***	5.6 ± 2.8	6.5 ± 1.6	6.3 ± 2.1	0.185
**∑PCBs, Male**	4.73 ± 3.14^a^	6.70 ± 1.41^b^	5.84 ± 1.90^ab^	0.032
**∑PCBs, Female**	6.35 ± 2.28	6.19 ± 1.85	6.88 ± 2.15	0.570
**PCB classifications**				
Wolff 2a (105, 118)*	37.1	37.1	36.0	0.992
Wolf 2b (138, 170)*	57.1	74.3	74.0	0.187
Wolf 3 (99, 153, 180, 183)*	34.3	57.1	50.0	0.144
McFarland-1b (105, 118, 138, 170)*	68.6	80.0	84.0	0.227
McFarland-2 (99, 101, 153, 180, 183)*	34.3^a^	65.7^b^	52.0^ab^	0.031
McFarland-3 (52, 151, 177, 187)*	71.4	80.0	80.0	0.593
Cooke-e (52, 95, 99, 101, 105, 110, 153)*	57.1	68.6	62.0	0.611
Kannan-1 (153, 180, 183, 187)*	37.1	45.7	40.0	0.757
Kannan-2a (52, 101, 110)*	22.9	20.0	22.0	0.956
Kannan-2b (95, 149, 151)*	88.6	94.3	90.0	0.685
Kannan-2b (95, 149, 151)**	2.21 (1.76–3.94)	2.47 (1.77–332)	2.25 (1.72–3.92)	0.936
Kannan_3a (105, 118)*	37.1	37.1	36.0	0.992
Kannan-3b (99, 138, 170, 177)*	62.9	85.7	80.0	0.060
Kannan-3b, Male*	47.1^a^	84.2^b^	75.0^ab^	0.040
Kannan-3b, Female*	77.8	87.5	86.4	0.686
Combined-1 (105, 110, 118)*	37.1	37.1	36.0	0.992
Combined-2 (138, 170, 177)*	62.9	82.9	78.0	0.126
Combined-3 (52, 95, 99, 101, 110, 149, 151, 153, 177, 180, 183, 187)*	94.3	100	100	
Combined-3***	3.29 ± 2.03	3.75 ± 1.46	3.64 ± 1.60	0.480
Ortho-substituted PCBs				
Monoortho (28, 105, 118)*	37.1	37.1	36.0	0.992
Diortho (52, 99, 101, 110, 138, 146, 153, 170, 180)*	85.7	100.0	100.0	
Diortho**	1.84 (1.42–3.53)	3.09 (2.41–3.79)	2.98 (1.84–3.93)	0.136
Diortho, male***	2.08 ± 1.70^a^	3.31 ± 1.23^b^	2.82 ± 1.16^ab^	0.028
Diortho, female***	3.06 ± 1.94	3.23 ± 1.56	3.33 ± 1.45	0.874
Triortho (95, 149, 151, 177, 183, 187)*	88.6	97.1	100.0	
Triortho**	2.45 (1.78–3.96)	2.68 (2.01–3.79)	2.61 (1.97–3.96)	0.780
Total chlorination				
Pentachlorinated (95, 99, 101, 105, 110,118)*	54.3	60.0	56.0	0.883
Hexachlorinated (138, 146, 149, 151, 153)*	94.3	100.0	98.0	
Hexachlorinated***	4.41 ± 2.36	4.98 ± 1.30	4.90 ± 1.96	0.398
Hexachlorinated, male***	3.64 ± 2.38^a^	5.20 ± 1.03^b^	4.56 ± 1.82^ab^	0.041
Hexachlorinated, female***	5.14 ± 2.15	4.71 ± 1.55	5.34 ± 2.08	0.619
Heptachlorinated (170, 177, 180, 183, 187)*	37.1	60.0	54.0	0.135

Values are given as %* or ng/g [median (25-75p)** and mean ± SD***]

*AGA* appropriate for gestational age, *CHLs* chlordanes, *DDD* dichlorodiphenyldichloroethane, *DDE* dichlorodiphenyldichloroethylene, *DDT* dichlorodiphenyltrichloroethane, *HCB* hexachlorobenzene, *HCCPDs* hexachlorocyclopentadienes, *HCH* hexachlorocyclohexane, *OCPs* organochlorine pesticides, *PCBs* polychlorinated biphenyls, *SGA* small for gestational age; ^ab^Values having different letters in the same row are statistically different (*p*<0.05)

The detection rates of PCBs and the mean level of ∑PCBs were similar across the groups. However, the mean levels of ∑PCBs, Diortho-substituted PCBs and Hexachlorinated PCBs were lower in preterm + AGA than term + SGA in male infants (*p* = 0.032, p = 0.028, *p* = 0.041; respectively, Fig. 1). The detection rates of Kannan-3b PCBs in male infants and McFarland-2 PCBs were lower in preterm + AGA than term + SGA (*p* = 0.040, *p* = 0.031, Table 3).

When mother-infant characteristics were included, GLM analysis revealed significant differences in the levels of both ∑DDTs and ∑OCPs based on gestational time and birth weight; lowest in cord blood of term + SGA infants (Table 4).

**Table 4:** Interaction for mother-infant characteristics with ∑DDT and ∑OCP*

		∑DDT			∑OCP	
Variables	Mean	95% Wald CI	p	Mean	95% Wald CI	p
Mother’s age, yrs			0.234			0.458
Mother’s education			0.483			0.365
< 12 yr	24.3	19.8–28.9		38.4	31.0–45.8	
≥ 12 yr	22.3	18.1–26.5		34.1	27.3–41.0	
Mother’s occupation			0.560			0.657
Housewife	22.2	19.0–25.4		34.9	29.7–40.1	
With job	24.4	18.2–30.6		37.6	27.5–47.8	
BMI before pregnancy, kg/m^2^			0.560			0.716
Weight gain during pregnancy			0.634			0.455
Low	24.7	20.3–29.1		38.4	31.6–45.6	
Appropriate	22.0	17.0–26.9		32.8	24.7–41.0	
High	23.3	18.9–27.6		37.5	30.4–44.6	
Smoke exposure			0.382			0.481
No	24.3	20.4–28.2		37.5	31.2–43.9	
Yes	22.3	18.3–26.4		35.0	28.3–41.6	
Type of birth			0.742			0.625
Natural	22.9	18.1–27.7		35.3	27.4–43.1	
Cesarean delivery	23.7	20.4–27.1		37.3	31.8–42.7	
Parity			0.438			0.453
First child	22.3	17.8–26.7		34.6	27.4–41.8	
≥ 2nd children	24.3	20.3–28.4		37.9	31.3–44.5	
Gender			0.902			0.823
Male	23.4	19.7–27.2		36.7	30.5–42.8	
Female	23.2	19.0–27.3		35.9	29.1–42.6	
Gestational time and birth weight			< 0.001			0.001
Preterm + AGA	24.5^a^	19.7–29.3		39.6^a^	31.8–47.5	
Term + SGA	17.3^b^	12.6–22.0		26.6^b^	18.9–34.4	
Term + AGA	28.1^a^	23.7–32.5		42.5^a^	35.3–49.7	

*AGA* appropriate for gestational age, *DDT* dichlorodiphenyltrichloroethane, *OCPs* organochlorine pesticides, *SGA* small for gestational age, *CI* confidence interval

^*^Generalized linear models analyzed the interaction for ∑DDT and ∑OCP with mother-infant variables. ^ab^Values having different letters in the same column are statistically different (*p*<0.05)

However, GLM analysis demonstrated no change for ∑PCBs with gestational time and birth weight, besides high weight gain during pregnancy, being the second child, and being a male infant were associated with lower PCB levels compared to their counterparts (Table 5). Upon conducting separate analyses by gender, it was found that in male infants, maternal exposure to cigarette smoke, first pregnancies, and term births were associated with higher PCB levels. However, the levels of PCBs in female infants did not show variations based on the studied characteristics. Among PCB classifications, diortho-substituted and combined-3 PCBs showed no differences with gestational age and birth weight.

**Table 5:** Interaction for mother-infant characteristics with PCBs, generalized linear models*

	∑PCB			Combined-3 PCBs			Diortho-substituted PCBs			∑PCB for male infants			∑PCB for female infants		
Variables	Mean	95% Wald CI	*p*	Mean	95% Wald CI	*p*	Mean	95% Wald CI	*p*	Mean	95% Wald CI	*p*	Mean	95% Wald CI	*p*
Mother’s age, yrs			0.877			0.333			0.304			0.619			0.898
Mother’s education			0.298			0.967			0.205			0.617			0.256
< 12 yr	6.4	5.7–7.2		3.7	3.1–4.4		3.0	2.4–3.5		6.0	5.0–6.9		6.9	5.6–8.1	
≥ 12 yr	5.9	5.2–6.6		3.8	3.2–4.3		2.5	2.1–3.0		5.7	4.8–6.6		5.9	4.9–7.0	
Mother’s occupation			0.662			0.515			0.450			0.757			0.653
Housewife	6.0	5.5–6.6		3.6	3.2–4.0		2.9	2.6–3.3		5.7	5.0–6.4		6.2	5.4–7.0	
With job	6.3	5.3–7.4		3.9	3.1–4.8		2.6	1.9–3.3		5.9	4.7–7.2		6.6	4.9–8.3	
BMI before pregnancy			0.630			0.404			0.442			0.607			0.259
Weight gain during pregnancy			0.008			0.808			0.011			0.062			0.062
Low	6.8^a^	6.1–7.6		4.2	3.6–4.8		3.2^a^	2.7–3.8		6.5	5.5–7.5		7.1	6.1–8.1	
Appropriate	6.2^ab^	5.4–7.1		3.9	3.2–4.6		2.7^ab^	2.1–3.2		6.0	4.9–7.0		6.4	5.1–7.7	
High	5.5^b^	4.8–6.2		3.1	2.6–3.7		2.4^b^	1.9–2.8		5.0	4.1–6.0		5.7	4.5–6.8	
Smoke exposure			0.126			0.578			0.150			0.017			0.608
No	5.9	5.2–6.5		3.7	3.1–4.2		2.6	2.1–3.0		5.2	4.3–6.1		6.5	5.6–7.5	
Yes	6.5	5.8–7.1		3.8	3.3–4.4		2.9	2.5–3.4		6.4	5.6–7.3		6.3	5.2–7.3	
Type of birth			0.081			0.326			0.011			0.370			0.139
Natural	5.8	5.0–6.6		3.6	2.9–4.2		2.4	1.8–2.9		5.9	4.6–6.6		5.9	4.7–7.2	
Cesarean delivery	6.5	6.0–7.1		3.9	3.5–4.4		3.1	2.7–3.5		6.1	5.4–6.8		6.9	6.0–7.7	
Parity			0.008			0.065			0.007			0.030			0.220
First child	6.8	6.0–7.5		4.1	3.5–4.7		3.2	2.7–3.7		6.4	5.5–7.3		6.8	5.6–8.0	
≥ 2nd children	5.6	4.9–6.3		3.4	2.9–4.0		2.3	1.9–2.8		5.2	4.4–6.1		6.0	5.0–7.0	
Gender			0.034			0.193			0.100						
Male	5.8	5.2–6.4		3.6	3.1–4.1		2.5	2.1–3.0							
Female	6.6	5.9–7.3		3.9	3.4–4.5		3.0	2.5–3.4							
Gestational time and birth weight			0.135			0.533			0.160			0.030			0.214
Preterm + AGA	5.7	4.9–6.5		3.5	2.9–4.1		2.4	1.8–2.9		4.8	3.7–5.8^a^		6.5	5.3–7.7	
Term + SGA	6.3	5.5–7.1		3.8	3.2–4.5		2.9	2.3–3.4		6.4	5.4–7.4^b^		5.8	4.5–7.0	
Term + AGA	6.6	5.9–7.3		3.9	3.3–4.5		3.0	2.5–3.5		6.3	5.3–7.3^b^		7.0	5.8–8.1	

*AGA* appropriate for gestational age, *PCBs* polychlorinated biphenyls, *SGA* small for gestational age, *CI* confidence interval

^*^Generalized linear models analyzed the interaction for PCBs with mother-infant variables. ^ab^Values having different letters in the same column are statistically different (*p*<0.05)

**Fig. 2:**
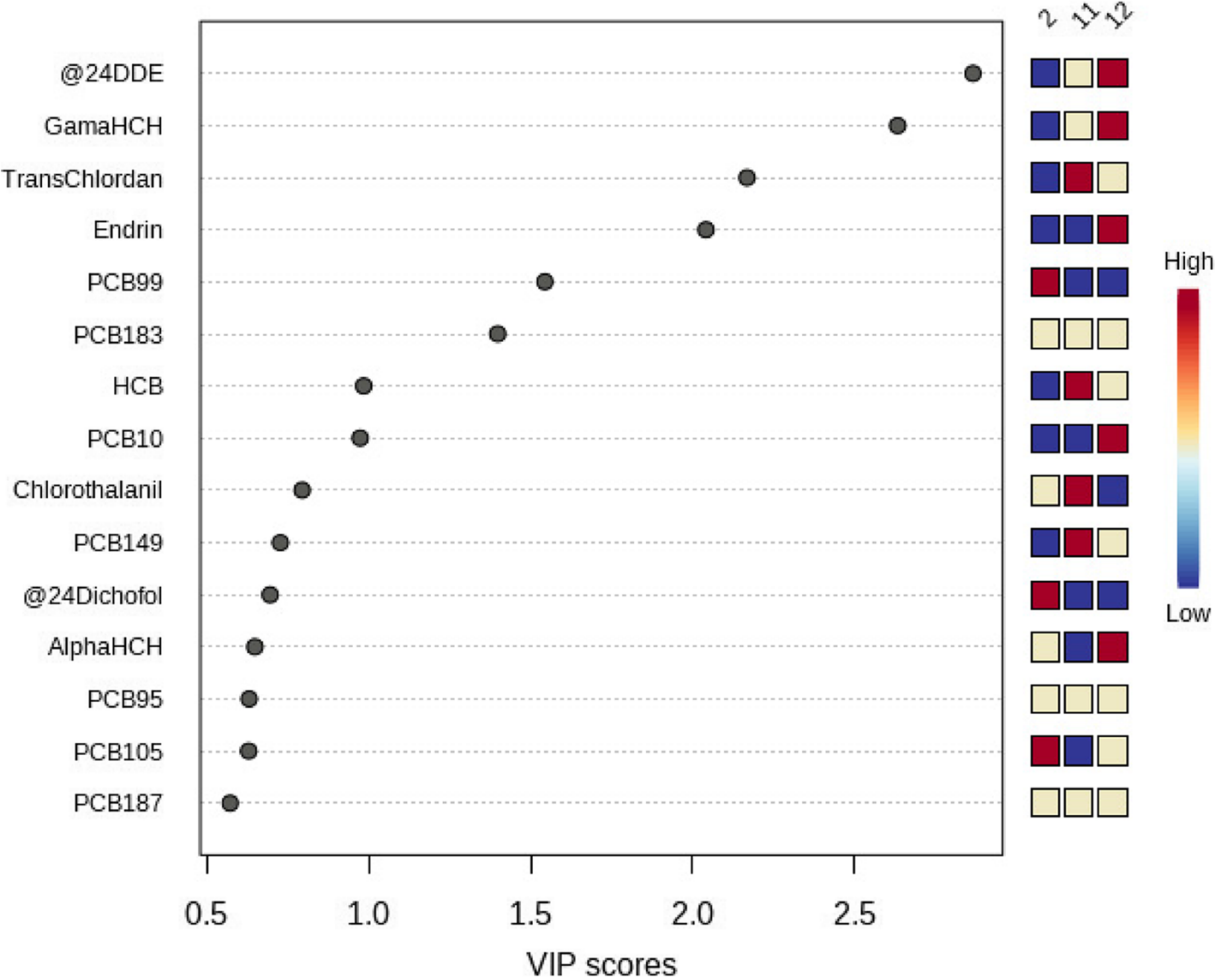
Variable importance in projection (VIP) scores of 15 top chemicals with Partial Least Squares Discriminant Analysis [Code 2: term + SGA; Code 11:preterm + AGA; Code 12:term + AGA]

PLS-DA showed the top VIP scores for 2,4’-DDE, gamma-HCH which were highest in term + AGA group (Fig. 2).

## Discussion

Numerous chemical compounds from both OCPs and PCBs were detected in most cord samples in Şanlıurfa, Turkey. In a previous study, β-HCH was reported as the predominant contaminant among the HCH compounds with newborn blood samples in İstanbul in 2011, but in our study, all samples were below the detection limit for β-HCH in cord blood samples. While the most common DDT pesticide in İstanbul was 4,4'-DDE, detected in 81.1% of newborns, in our study, 2,4'-DDE (79.2%) was the most prevalent, with 4,4'-DDE detected in only 9.2% of cord blood samples. The detection rate for 4,4'-DDT in Istanbul was 21.6%, whereas in Şanlıurfa, it was 40.8%. oxy-chlordane was detected in 37.8% of samples in İstanbul but was not detected in our study. The detection rate of cis-chlordane was only 5.4% in İstanbul compared to 23.3% in our study. Pesticides such as aldrin and endrin had detection rates of less than 10% in both studies. In İstanbul, HCB was detected in 48.6% of participants, whereas in our study, it was detected in 19.2%. Additionally, in İstanbul, at least one of the 18 PCB congeners studied was present in 62.2% of all blood samples, whereas in our study, at least one PCB congener was present in 98.3% of samples. These findings indicate that pollutant exposures vary according to geographical regions.” Was added to discussion section. Interestingly, irrespective of the gestational weeks, cord blood obtained from SGA infants consistently exhibited the lowest levels of OCPs. Upon closer examination, we observed that parity and gestational duration emerged as significant factors linked to PCB levels in the cord blood of male infants. In contrast, when considering female infants, cord PCB levels remained unaffected by maternal and infant characteristics. This underscores the complexity of the interactions between maternal and infant factors and their differential impacts on the presence of specific chemical compounds in cord blood. The distinct patterns observed in male and female infants suggest a need for further investigation into gender-specific vulnerabilities and responses to environmental exposures during gestation.

Similar to previous review in Eastern Europe [44], a 4,4’-DDT/4,4’-DDE ratio above 1 indicates continued recent exposure in the area. Our research reveals a noteworthy negative interaction between the cumulative levels of ∑OCPs and ∑DDTs and SGA among male infants. Notably, previous studies have already documented a positive correlation between the concentration of 4,4’-DDE in cord blood and birth weight [15]. Furthermore, there is existing evidence suggesting that elevated levels of 4,4’-DDE are associated with infant growth, manifesting as higher BMI at the age of 2 years [7, 18, 45]. However, a comprehensive meta-analysis conducted by Stratakis in 2022, which scrutinized the effect estimates for prenatal exposure to DDE and BMI-z during infancy (0–2 years), did not identify a significant association [46]. Interestingly, the landscape of findings varies across studies. For instance, one study reported a significant increase in BMI-z with prenatal exposure to DDE in girls but not in boys [7], while another study found a negative association between DDE and BMI-z at 6 months of age, specifically in boys [47]. These nuanced results underscore the complexity of the relationship between prenatal exposure to DDE and subsequent impacts on BMI, emphasizing the importance of considering gender-specific dynamics in future research endeavors.

In our investigation, PCB 151, PCB 149, PCB 138, and P146 emerged as the most prevalent congeners, each surpassing the 50.0% threshold. In prior reports PCB 138 and PCB 153 were typically identified as the primary contributors to PCB congeners [48–52]. The absence of PCB 28 in cord blood samples in our study is notable, as it fell below its respective limit of quantification (LOQ). It's worth noting that there is limited available data on PCB contaminants in various biological matrices in Turkey [25–28, 53]. Ulutaş et al. [25] discovered that PCB 153 (75%), PCB 180 (68%), and PCB 138 (66%) were the predominant contaminants in human blood samples, with PCB 153 being the most substantial contributor to the overall PCB content in İstanbul, Turkey. Previous studies reported PCBs in umbilical cord serum samples in other countries [50, 54, 55]. Despite the well-established reputation of PCBs for their prolonged half-life and propensity to accumulate in biological systems, certain studies have failed to establish a clear positive correlation between maternal age and PCB levels in umbilical cord blood [48]. Similarly, investigations into human milk have yielded inconclusive results regarding such an association [56]. Interestingly, our study diverges from these trends, revealing higher levels of PCBs in first pregnancies. This suggests a potential elimination of these compounds from the maternal body during subsequent pregnancies. In contrast, one study in Brazil did not identify a significant difference between primiparous (first-time mothers) and multiparous (those with multiple pregnancies) individuals [48]. This discrepancy highlights the complexity of factors influencing PCB dynamics in maternal and fetal compartments, urging further exploration into the nuanced interactions shaping the presence and elimination of PCBs during pregnancy and lactation.

Our investigation did not reveal any significant interaction between ∑PCB levels and AGA-SGA status. However, an interesting observation was made in the cord blood PCB levels of the preterm-AGA group, which were found to be lower than those in the term-sga group among male infants. No such difference was noted for term-AGA infants. This suggests that cord blood PCB transfer increases with gestational age, regardless of birth weight. Some studies have indicated higher odds of SGA with increasing levels of PCB 153 [23, 57, 58]. Unfortunately, most studies analyzed birth weight without considering gestational duration. The landscape of findings from previous studies on this subject is marked by inconsistency. Some studies report minimal or no associations between PCBs and birth weight [15, 59], while others even suggest inverse associations [23, 57, 60–62]. Govarts et al. [63] conducted a meta-analysis, revealing a neonatal weight decline of 150 g for each 1 μg/L increase in PCB 153. In Denmark, Kofoed et al. [13] reported a 32 g lower weight for children born to women exposed to PCB analytes compared to those born to unexposed women. In contrast, Lignell et al. [60] found that prenatal exposure to the sum of PCB 138, PCB 153, and PCB 180 was significantly associated with increased neonatal weight. More recently, a negative correlation was reported in Iran for PCB 52, where a one-unit increase in cord serum levels corresponded to a 0.024 g decrease in newborn weight [64]. In two studies, a reduction in birth weight was specifically noted in association with PCBs, but this effect was observed primarily among boys [65, 66]. Moreover, a meta-analysis encompassing seven studies, two of which involved cord blood levels, indicated a negative correlation between PCB exposure and birth weight [11]. However, prenatal exposure to the ΣPCBs had also been linked to higher BMI-z in infancy [46, 67], a negative association with the change in weight from birth to 3 months [68] and no association between PCB-153 and weight-for-age from birth to 24 months in a multi-center European study [18]. These varying outcomes may arise from differences in the specific PCB congener profiles studied, variations in exposure levels, and population characteristics.

In our study, male infants born at term with SGA status exhibited higher levels of di-ortho-substituted PCBs (including PCB 153) compared to male infants born preterm with AGA status. However, no significant association was found between tri-ortho-substituted PCBs (including PCB 187) and the risk of preterm birth or low birth weight. On contrary, Patel et al. [61] reported specific associations between maternal serum PCB levels (PCB 18, PCB 153, and PCB 187) and birth weight, with each 10 ng/g lipid increase linked to varying decreases in birth weight (a 138.4 g, 41.9 g and 170.4 g lower, respectively) for girls with mothers in the lowest educational level. Interestingly, Lamb et al. [69] revealed an association between maternal levels of ortho-substituted PCBs and reduced weight throughout 17 years of age in girls, besides an interaction between tri-ortho-substituted PCBs and increased height in boys [69]. These intriguing patterns suggest gender-specific effects and underscore the importance of considering not only the overall PCB exposure but also the specific congener profiles when assessing their impact on fetal growth and development.

In our study, PLS-DA unveiled that 2,4’-DDE, gamma-HCH, transchlordan, endrin, and PCB 99 exhibited notable differences when comparing infants categorized as AGA versus SGA, as well as those born at term versus preterm. It is noteworthy that, two studies [23, 70] have employed a multi-pollutant regression model, incorporating various PCB congeners, 4,4’-DDE, and HCB, to assess the association between multiple exposures and birth weight. This approach reflects a comprehensive perspective, recognizing the intricate interplay of different pollutants in influencing birth outcomes. The limited number of studies employing such a multi-pollutant regression model underscores the complexity of evaluating the combined effects of diverse environmental exposures on fetal development and highlights the potential for future research to explore these interactions more comprehensively.

### Limitation and strengths

This study is subject to certain limitations that warrant consideration. One notable constraint is the reliance on a single exposure assessment conducted at the time of delivery. This approach may not capture potential variations in exposure levels throughout the entire gestational period. However, given the persistent nature of the exposure under investigation, the chosen assessment method reasonably reflects prenatal exposure. Moreover, the study depends on maternal self-reporting to identify behavioral risks such as smoking and drug use. This reliance introduces a potential source of error, as self-reported data are susceptible to recall bias and social desirability bias. These biases could impact the accuracy and completeness of the information collected, potentially influencing the reliability of the associations drawn from these reported behaviors.

Lipids were not measured and the lipid adjustment cannot be performed in this study. However, QuEChERS technique, the extraction method employed, ensures the efficient and precise isolation of target compounds from umbilical plasma. The QuEChERS technique is not typically adjusted for lipid extraction due to the inherent nature of its design [36, 37, 71, 72]. The analytical approach applied to the samples, coupled with its stringent calibration and performance validation, inspires confidence in the accuracy and reproducibility of the study's results. The use of cutting-edge equipment and strict adherence to standardized protocols within the laboratory significantly contribute to the robustness of the chromatographic analysis.

The study benefits from the low-risk antenatal profiles of the women participants. This characteristic serves as a strength by minimizing other potential competing risk factors that could influence altered fetal growth. In many studies, low birth weight (< 2500 g) has been used to assess the interaction with POPs. However, this approach can lead to inaccurate assessments for infants with appropriate gestational weight gain. Therefore, we standardized birth weight with gestational age to mitigate this issue in our study.

For pollutants detected between 10 and 90% of samples, the frequency of being above the detection limit was compared. It was noted that if the rate of being below the detection limit is less than 30%, due to the limitation of a small number of cases, some significances may not be determined. Therefore, the analysis of these pollutants will necessitate larger datasets in future studies.

## Conclusion

The analysis of cord blood samples revealed the presence of numerous OCPs and PCBs. Notably, cord blood obtained from SGA infants consistently exhibited the lowest levels of OCPs in overall group and male infants, and preterm birth showed lowest PCBs in male infants. Upon closer scrutiny, significant associations were identified particularly in male infants. The distinct gender-specific patterns observed in male and female infants underscore the complexity of environmental exposures during gestation and suggest potential gender-specific vulnerabilities. Further investigation into these gender-specific responses is warranted to deepen our understanding of the differential impacts of prenatal exposures on male and female infants.

### Supplementary Information

Supplementary Material 1.

## Data Availability

Data can be requested from corresponding author (siyalcin@hacettepe.edu.tr).
