# Supplementary material for: Exploring the levels of persistent organic pollutants in umbilical cord blood and their connection to gestational age and birth weights in Şanlıurfa, Turkey
Source: BMC Pregnancy Childbirth. 2024 Jul 25;24:501. doi: 10.1186/s12884-024-06677-8 (PMC11270763; doi:10.1186/s12884-024-06677-8)
Supplement: Supplementary file 1 — Supplementary Material 1. [file 12884_2024_6677_MOESM1_ESM.docx]

**Exploring the levels of persistent organic pollutants in umbilical cord blood and their connection to gestational age and birth weights in Şanlıurfa, Turkey**

Suplementary Table. Quality assurance parameters of selected ion monitoring (SIM) method at gas chromatograph/mass spectrometry

|  | Purity (%) | R^2^ | Recovery, % | LOD, ng/g | LOQ, ng/g | Reference Ions | Retention Time (Min.) | u (x); k = 2 |
| --- | --- | --- | --- | --- | --- | --- | --- | --- |
| alpha-HCH | 99.50 | 0.998 | 97.7 | 0.23 | 0.69 | 181, 183, 219 | 8.8 | 0.065 |
| beta-HCH | 99.20 | 0.998 | 93.0 | 0.31 | 0.93 | 284, 286, 282 | 8.9 | 0.093 |
| delta-HCH | 99.70 | 0.998 | 104.9 | 0.18 | 0.54 | 181, 183, 219 | 10.3 | 0.054 |
| gama-HCH | 98.50 | 0.999 | 97.9 | 0.17 | 0.51 | 181, 183, 219 | 9.6 | 0.101 |
| 2,4’-DDD | 98.50 | 0.998 | 100.8 | 0.18 | 0.54 | 235, 237, 165 | 15.4 | 0.085 |
| 4,4’-DDD | 99.50 | 0.998 | 104.7 | 0.19 | 0.57 | 235, 237, 165 | 16.5 | 0.034 |
| 2,4’-DDE | 99.00 | 0.998 | 98.8 | 0.20 | 0.60 | 246, 248, 318 | 14.3 | 0.048 |
| 4,4’-DDE | 98.50 | 0.998 | 97.4 | 0.18 | 0.54 | 246, 318, 218 | 15.2 | 0.055 |
| 2,4’-DDT | 99.50 | 0.998 | 105.4 | 0.20 | 0.60 | 235, 237, 165 | 16.6 | 0.110 |
| 4,4’-DDT | 98.50 | 0.998 | 101.0 | 0.16 | 0.48 | 235, 237, 165 | 17.6 | 0.073 |
| oxy-Chlordan | 99.50 | 0.998 | 97.4 | 0.21 | 0.63 | 115, 185, 187 | 13.6 | 0.068 |
| cis-Chlordan | 99.00 | 0.998 | 96.2 | 0.21 | 0.63 | 373, 375, 377 | 14.2 | 0.035 |
| trans-Chlordan | 99.90 | 0.998 | 98.8 | 0.43 | 1.29 | 373, 375, 377 | 14.6 | 0.049 |
| Chlorothalonil | 98.50 | 0.999 | 104.9 | 0.18 | 0.54 | 266, 264, 268 | 10.0 | 0.051 |
| Aldrin | 99.00 | 0.998 | 95.1 | 0.22 | 0.66 | 66, 263, 91 | 12.5 | 0.088 |
| Endrin | 99.00 | 0.999 | 99.3 | 0.14 | 0.42 | 81, 263, 265 | 16.0 | 0.060 |
| Heptachlor | 99.00 | 0.999 | 100.5 | 0.16 | 0.48 | 100, 272, 274 | 11.5 | 0.091 |
| Endosulfan-sulfate | 99.00 | 0.998 | 101.3 | 0.17 | 0.51 | 272, 229, 387 | 17.5 | 0.059 |
| Total Dicofol (2,4’) | 99.90 | 0.999 | 101.0 | 0.38 | 1.14 | 139, 250, 11 | 12.4 | 0.042 |
| Hexachlorobenzene (HCB) | 99.50 | 0.998 | 98.4 | 0.23 | 0.69 | 284, 286, 282 | 8.9 | 0.054 |
| PCB28 | 99.20 | 0.999 | 99.6 | 0.10 | 0.30 | 256, 258, 186 | 11.8 | 0.070 |
| PCB52 | 99.60 | 0.999 | 99.9 | 0.12 | 0.37 | 292, 220, 200 | 12.9 | 0.092 |
| PCB95 | 96.00 | 0.999 | 100.5 | 0.13 | 0.39 | 326, 254, 256 | 14.9 | 0.043 |
| PCB99 | 98.90 | 0.999 | 101.1 | 0.11 | 0.33 | 326, 254, 256 | 15.7 | 0.108 |
| PCB101 | 97.30 | 0.999 | 101.6 | 0.17 | 0.51 | 326, 254, 256 | 15.8 | 0.120 |
| PCB105 | 99.50 | 0.999 | 100.8 | 0.14 | 0.42 | 326, 328, 254 | 16.8 | 0.039 |
| PCB110 | 97.80 | 0.999 | 104.6 | 0.15 | 0.45 | 360, 290, 362 | 17.2 | 0.065 |
| PCB118 | 99.50 | 0.999 | 102.2 | 0.19 | 0.57 | 360, 290, 362 | 17.6 | 0.081 |
| PCB138 | 98.80 | 0.999 | 102.6 | 0.14 | 0.42 | 326, 328, 324 | 17.8 | 0.026 |
| PCB146 | 97.60 | 0.999 | 102.6 | 0.24 | 0.72 | 360, 362, 290 | 18.3 | 0.056 |
| PCB149 | 99.90 | 0.999 | 102.6 | 0.20 | 0.60 | 360, 362, 290 | 18.6 | 0.062 |
| PCB151 | 98.50 | 0.999 | 102.6 | 0.24 | 0.72 | 326, 328, 324 | 18.7 | 0.100 |
| PCB153 | 98.80 | 0.999 | 102.1 | 0.18 | 0.54 | 360, 362, 290 | 19.5 | 0.045 |
| PCB170 | 93.60 | 0.999 | 102.5 | 0.19 | 0.57 | 396, 394, 324 | 20.0 | 0.077 |
| PCB177 | 99.30 | 0.999 | 94.9 | 0.21 | 0.63 | 396, 394, 324 | 20.2 | 0.058 |
| PCB180 | 97.90 | 0.999 | 95.4 | 0.22 | 0.66 | 396, 394, 324 | 21.0 | 0.066 |
| PCB183 | 97.00 | 0.999 | 94.3 | 0.16 | 0.48 | 396, 394, 324 | 21.9 | 0.054 |
| PCB187 | 99.70 | 0.999 | 99.3 | 0.23 | 0.69 | 396, 394, 324 | 22.9 | 0.071 |

LOD: limit of detection; LOQ: limit of quantification; CHLs: chlordanes; DDD: dichlorodiphenyldichloroethane; DDE: dichlorodiphenyldichloroethylene; DDT: dichlorodiphenyltrichloroethane; HCB: hexachlorobenzene; HCCPDs: hexachlorocyclopentadienes; HCH: hexachlorocyclohexane; OCPs: organochlorine pesticides; PCBs: polychlorinated biphenyls;
